# Preventive Effect of *Lactobacillus helveticus* SBT2171 on Collagen-Induced Arthritis in Mice

**DOI:** 10.3389/fmicb.2017.01159

**Published:** 2017-06-21

**Authors:** Maya Yamashita, Kurumi Matsumoto, Tsutomu Endo, Ken Ukibe, Tomohiro Hosoya, Yumi Matsubara, Hisako Nakagawa, Fumihiko Sakai, Tadaaki Miyazaki

**Affiliations:** ^1^Milk Science Research Institute, Megmilk Snow Brand Co., Ltd.Saitama, Japan; ^2^Department of Orthopedic Surgery, Graduate School of Medicine, Hokkaido UniversitySapporo, Japan; ^3^Department of Probiotics Immunology, Institute for Genetic Medicine, Hokkaido UniversitySapporo, Japan

**Keywords:** *Lactobacillus helveticus* SBT2171, rheumatoid arthritis, collagen-induced arthritis, autoantibody production, pro-inflammatory cytokines

## Abstract

We recently reported that the intraperitoneal inoculation of *Lactobacillus helveticus* SBT2171 inhibited the development of collagen-induced arthritis (CIA), a murine model of rheumatoid arthritis (RA). In the present study, we evaluated the effect of the oral administration of *L. helveticus* SBT2171 on CIA development and on the regulation of antigen-specific antibody production and inflammatory immune cells, which have been implicated in the development of RA. Both oral administration and intraperitoneal inoculation of *L. helveticus* SBT2171 reduced joint swelling, body weight loss, and the serum level of bovine type II collagen (CII)-specific antibodies in the CIA mouse model. The intraperitoneal inoculation also decreased the arthritis incidence, joint damage, and serum level of interleukin (IL)-6. In addition, the numbers of total immune cells, total B cells, germinal center B cells, and CD4^+^ T cells in the draining lymph nodes were decreased following intraperitoneal inoculation of *L. helveticus* SBT2171. These findings demonstrate the ability of *L. helveticus* SBT2171 to downregulate the abundance of immune cells and the subsequent production of CII-specific antibodies and IL-6, thereby suppressing the CIA symptoms, indicating its potential for use in the prevention of RA.

## Introduction

Lactic acid bacteria (LAB) is a general term used to represent bacteria that produce lactic acid by metabolism. LAB have long been used for the fermentation of foods such as yogurt, *Lactobacillus*-based beverages, and pickles, and contribute to improving the shelf-life, nutritive value, and palatability of foods, leading to the enrichment of consumer diets. Some strains of LAB naturally exist in the human gastrointestinal tract, including the intestine, as well as in the vagina, and are known to be useful for homeostasis maintenance of the gut environment that is compromised by rivaling pathogenic microorganisms (Parvez et al., 2006; Murooka and Yamshita, 2008; Kabak and Dobson, 2011).

Recently, the healthy function of LAB has been reported to not only improve the environment of the intestine but to also have an influence on immune functions. For example, oral administration of *Lactobacillus gasseri* SBT2055 to mice was found to be effective in the protection against influenza virus infection via suppressing viral replication through the induction of the expression of antiviral genes (Nakayama et al., 2014). In addition, *Bifidobacterium longum* BB536 was shown to augment the activity of natural killer cells, a type of cytotoxic lymphocyte that is critical for the proper function of the innate immune system, and exerted preventive effects against influenza virus infection in elderly people (Namba et al., 2010; Akatsu et al., 2013). *Lactobacillus rhamnosus* GG-derived protein promotes IgA production, which plays a critical role in immune function, including host defense against infection and oral tolerance in the mucous membranes, in the intestinal epithelial cells of mice (Wang et al., 2016). Moreover, although there are relatively few reports on the immune-promoting properties of LAB, they have been shown to both downregulate and activate components of the immune system. These reports have demonstrated that LAB have an alleviative effect on allergy (Shandilya et al., 2016; Yamamoto et al., 2016) and autoimmune diseases (Kato et al., 1998; So et al., 2008; Amdekar et al., 2011) resulting from an excessive immune system response due to mistaking originally harmless triggers such as food, pollen, and self-proteins as harmful. However, the specific mechanisms by which LAB exert these effects remain unclear.

To contribute toward resolving these issues, in this study, we focused on *Lactobacillus helveticus* SBT2171, a strain of lactobacilli that is derived from dairy products, possesses high protease activity, and is commonly used as a starter bacterial strain in the production of a Gouda-type cheese (Sasaki et al., 1996). Our previous study clarified that *L. helveticus* SBT2171 significantly suppressed the proliferation of primary murine immune cells among 41 LAB strains of various species, and decreased the production of lipopolysaccharide (LPS)-stimulated inflammatory cytokines [interleukin (IL)-6 and IL-1β] from the immune cells *in vitro* (Yamashita et al., 2014). In addition, *in vivo* infection of *L. helveticus* SBT2171 prevented the development of rheumatoid arthritis (RA) in a murine model (Hosoya et al., 2014), one of the major autoimmune diseases. This murine model is commonly used in research on RA, and is established by the subcutaneous injection of adjuvant-activated collagen, leading to collagen-induced arthritis (CIA) that shows RA-like symptoms such as swelling and degeneration in a joint and disturbance of gait (Billiau and Matthys, 2011). Our previous study (Hosoya et al., 2014) demonstrated that the intraperitoneal inoculation of *L. helveticus* SBT2171, before the onset of CIA, reduced the incidence and clinical score of CIA compared to control mice.

In the present study, to clarify these potential beneficial properties of *L. helveticus* SBT2171 in more detail, we investigated its effects on factors that are critically involved in the development and progression of CIA, including antibody titer, pro-inflammatory cytokines, and behavior of immune cells, and compared these effects with two administration routes: oral administration and intraperitoneal inoculation. These findings can help to further elucidate the mechanisms by which LAB promote immune function and should contribute to the promotion and development of LAB-based treatments for immunological diseases.

## Materials and Methods

### Bacterial Strain

*Lactobacillus helveticus* SBT2171 was isolated by Megmilk Snow Brand (Tokyo, Japan), and inoculated into Lactobacilli MRS broth (BD Biosciences, San Jose, CA, United States) and cultivated for 16 h at 37°C. Following incubation, the cells were harvested by centrifugation at 8,000 × *g* for 10 min. The cells were washed twice with saline and once with distilled water and freeze-dried.

For intraperitoneal inoculation, the freeze-dried bacterial cells were resuspended in phosphate-buffered saline (PBS) at 10 mg/mL and heat-killed at 80°C for 30 min. For oral administration, the freeze-dried bacterial cells (2 × 1.10^10^ cfu/g in powder form) were resuspended in 0.35 M NaHCO_3_ in PBS at 100 mg/mL.

### Antibodies

The following fluorochrome-labeled anti-mouse antibodies were used for flow cytometry with fluorescence-activated cell sorting (FACS) analyses: anti-B220 (RA3-6B2), anti-CD138 (281-2), anti-GL7 (GL7), anti-CD3 (145-2C11), anti-CD4 (GK1.5), anti-CD8 (53-6.7), anti-IL-17A (TC11-18H10.1), anti-IFN-γ (XMG1.2), anti-PD-1 (RMP1-30), anti-CXCR5 (L138D7) (BioLegend, San Diego, CA, United States); anti-CD25 (3C7) (BD Biosciences); and anti-Foxp3 (FJK-16s) (eBioscience, San Diego, CA, United States). Biotinylated anti-mouse antibodies anti-IgG1 (RMG1-1) and anti-IgG2a (RMG2a-62) (BioLegend), and a peroxidase-conjugated anti-mouse IgG polyclonal antibody (Jackson ImmunoResearch, West Grove, PA, United States) were used for enzyme-linked immunosorbent assay (ELISA).

### Mice

DBA/1J mice (Japan SLC, Shizuoka, Japan) received sterile water and standard chow (Labo MR stock; Nosan Corporation, Yokohama, Japan) *ad libitum*. All experiments involving mice were carried out in accordance with the guidelines of the Bioscience Committee of Hokkaido University and were approved by the Animal Care and Use Committee of Hokkaido University.

### Induction of CIA and Oral Administration and Inoculation of *L. helveticus* SBT2171

Male DBA/1J mice (8 weeks old) were used to assess the immunosuppressive effect of *L. helveticus* SBT2171 on CIA in mice (*n* = 8–12). To induce CIA (Billiau and Matthys, 2011; Kanayama et al., 2011; Hosoya et al., 2014), the mice were immunized by intradermal injection at the base of the tail with 100 μg of bovine type II collagen (CII; Chondrex, Redmond, WA, United States), which was dissolved in 50 μL of 0.05 M acetic acid and emulsified in 50 μL of Complete Freund’s Adjuvant (CFA; Chondrex) containing 250 μg of heat-killed *Mycobacterium tuberculosis* H37RA. At day 21 after the first immunization, the mice were re-immunized with CII emulsified in CFA. The CIA mice were divided into two groups: (1) mice receiving *L. helveticus* SBT2171 and (2) mice receiving vehicle only (control).

For the assessment of the intraperitoneal inoculation of *L. helveticus* SBT2171, the mice were injected with 100 μL of PBS containing 1 mg of freeze-dried and heat-treated bacterial cells intraperitoneally by a single injection three times per week after the first immunization, and then daily after the second immunization. Using the same treatment regimen, mice in the control group received the same volume of PBS only by intraperitoneal injection. For oral administration of *L. helveticus* SBT2171, 300 μL of an NaHCO_3_/PBS solution containing 30 mg of freeze-dried bacterial cells (1.2 × 10^10^ cfu/g) was forcedly administered into the stomach with a feeding needle by single oral gavage daily after the first immunization. Using the same treatment regimen, mice in the control group received the same volume of NaHCO_3_/PBS only orally.

In both experiments, the incidence of CIA, disease severity, paw thickness, and body weight were assessed after the second immunization for 21 days. The arthritic disease severity in each paw was scored on a 0–4 scale as described by Kanayama et al. (2011) as follows: 0, normal; 1, focal slight swelling and/or redness in one digit; 2, moderate swelling and erythema of >2 digits; 3, marked swelling and erythema of the limb; and 4, maximal swelling, erythema, deformity, and/or ankylosis. The maximum possible score for each mouse was 16.

### Histopathological Assessment

The hind limb joint tissues were obtained on day 42 from the mice with CIA (*n* = 4; they were selected in a random manner). The tissues were fixed in 10% paraformaldehyde, decalcified in ethylenediaminetetraacetic acid (Sigma-Aldrich, Tokyo, Japan), and embedded in paraffin. The samples were prepared and stained with hematoxylin and eosin (H&E; Merck Millipore, Guyancourt, France) or Safranin O (WALDECK GmbH & Co Division Chroma, Münster, Germany). H&E-stained sections were used to evaluate the degree of pannus formation (scored from 0 to 4 as follows: 0, normal; 1, minor leukocyte infiltration into the synovium; 2, mild synovium outgrowth; 3, synovium invasion into the joint space, and 4, fibrous ankylosis of the joints). Safranin O-stained sections were used to evaluate the degree of cartilage degeneration (scored from 0 to 4 as follows: 0, normal; 1, minor erosion of the cartilage; 2, mild erosion of the cartilage; 3, partial erosion of the subchondral cartilage; and 4, erosion in all layers of the subchondral cartilage). The average score obtained from the two hind limbs was determined as the score for each mouse, with a maximum possible score of 4.

### Measurement of Collagen-Specific and Total IgG Levels

Serum of the CIA mice was collected on day 42 after the first immunization. The level of total type II collagen-specific IgG (CII-IgG) and its subclasses, CII-IgG1 and CII-IgG2a, were measured by ELISA. In brief, immunoplates (Nunc, Thermo Fisher Scientific, Hudson, NH, United States) were coated with a solution containing 5 mg/mL of CII (Chondrex) and incubated at 4°C overnight. After blocking with 1% bovine serum albumin in PBS, serum samples were added into the CII-coated wells and the plates were incubated for 2 h at room temperature (RT). After five washes with 0.05% Tween in PBS (Tween-PBS), the plates were incubated with horseradish peroxidase (HRP)-conjugated anti-mouse IgG antibody, biotinylated anti-mouse IgG1 antibody, or biotinylated anti-mouse IgG2a antibody for 1 h at RT. When the biotinylated secondary antibody was used, the plates were subsequently incubated with avidin-HRP solution (BioLegend) for 30 min at RT. Following five washes with Tween-PBS, the plates were developed with tetramethylbenzidine (Kirkegaard & Perry Laboratories, Gaithersburg, MD, United States) and the reaction was stopped by the addition of 1 N HCl. Absorbance values at 450 nm and 630 nm were measured. The total IgG level in the serum was evaluated using the Mouse IgG ELISA Quantitation Set (Bethyl Laboratories, Montgomery, TX, United States) according to the manufacturer’s instructions (Johansen et al., 1999).

### Measurement of Cytokine Levels

The levels of IL-6 and tumor necrosis factor-alpha (TNF-α) in the serum at day 42 after the first immunization were measured with the Mouse IL-6 ELISA MAX^TM^ Standard and Mouse TNF-α ELISA MAX^TM^ Standard kits (BioLegend) according to the manufacturer’s instructions (Patole et al., 2005; Stolk et al., 2006).

### Cell Staining and Flow Cytometric Analysis

At day 42 after the first CII immunization, the inguinal lymph nodes (LNs) of the mice were collected and mechanically disrupted in RPMI 1640 culture medium (Wako, Osaka, Japan) containing 10% heat-inactivated fetal bovine serum (GIBCO, Thermo Fisher Scientific, Waltham, MA, United States), 100 U/mL of penicillin, 100 μg/mL of streptomycin (Sigma-Aldrich, St. Louis, MO, United States), and 0.05 mM 2-mercaptoethanol. Cell suspensions were filtered through a 100-μm cell strainer (BD Biosciences), washed twice with the medium, and resuspended in the cell culture medium.

The following markers were used to determine the type of immune cells: B cells (B220^+^), germinal center (GC) B cells (GL7^+^ B220^+^), plasma cells (CD138^+^ B220^-^), CD4^+^ T cells (CD3^+^ CD4^+^), CD8^+^ T cells (CD3^+^ CD8^+^), Th1 cells (CD3^+^ CD4^+^ IFN-γ^+^), Th17 cells (CD3^+^ CD4^+^ IL-17A^+^), regulatory T cells (Tregs; CD4^+^ CD25^+^ Foxp3^+^), follicular T helper (Tfh) cells (CD4^+^ CXCR5^+^ PD-1^+^ Foxp3^-^), and follicular regulatory T cells (Tfrs; CD4^+^ CXCR5^+^ PD-1^+^ Foxp3^+^), and these molecular markers used in the FACS analysis are shown in **Table 1**.

**Table 1:** Molecular markers used in the FACS analysis.

Type of immune cells	Molecular markers
B cells	B220^+^
Germinal center B cells	GL7^+^ B220^+^
Plasma cells	CD138^+^ B220^-^
CD4^+^ T cells	CD3^+^ CD4^+^
CD8^+^ T cells	CD3^+^ CD8^+^
Th1 cells	CD3^+^ CD4^+^ IFN-γ^+^
Th17 cells	CD3^+^ CD4^+^ IL-17A^+^
Regulatory T cells	CD4^+^ CD25^+^ Foxp3^+^
Follicular T helper cells	CD4^+^ CXCR5^+^ PD-1^+^ Foxp3^-^
Follicular regulatory T cells	CD4^+^ CXCR5^+^ PD-1^+^ Foxp3^+^


For the Th1, Th17, Treg, and Tfr staining, the cells were stimulated with phorbol-12-myristate 13-acetate (50 ng/mL) and ionomycin (500 ng/mL) in the presence of brefeldin A (1 μL/mL) (GolgiPlug; BD Biosciences, Heidelberg, Germany) for 5.5 h. Subsequently, the cells were stained intracellularly with anti-IFN-γ, anti-IL-17A, and anti-Foxp3 antibodies using the BD Cytofix/Cytoperm reagents (BD Biosciences) or Foxp3 Fix/Perm buffer (BioLegend) according to the manufacturer’s protocols. Cytometric data were acquired using a FACSCanto II flow cytometer and analyzed with FACSDiva software (BD Biosciences).

### Statistical Analysis

For the disease severity score, paw thickness, and body weight change results, statistical analyses were performed using two-way repeated-measures analysis of variance. The log-rank test was used to assess differences in CIA incidence, and the Mann–Whitney *U*-test was used for other measured values. The log-rank test was performed using EZR (Saitama Medical Center, Jichi Medical University, Saitama, Japan), which is a graphical user interface for R (version 1.21; the R Foundation for Statistical Computing, Vienna, Austria). Other analyses were performed using StatView version 5.0 (SAS Institute, Cary, NC, United States). *P*-values of <0.05 were considered statistically significant.

## Results

### *Lactobacillus helveticus* SBT2171 Attenuated CIA-Associated Symptoms

**FIGURE 1:**
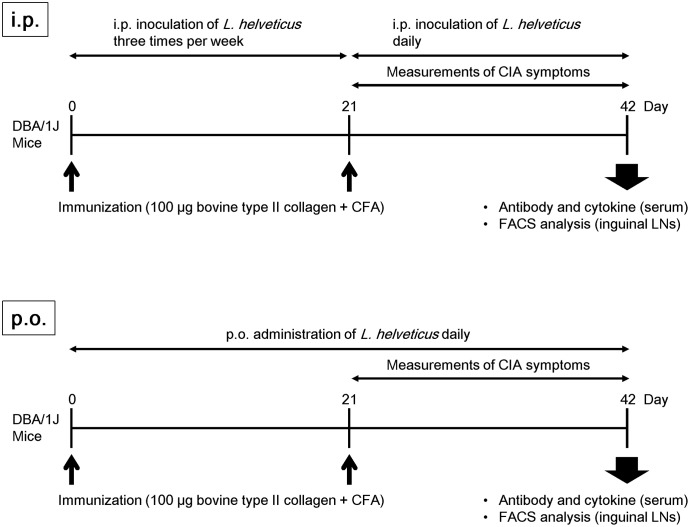
Experimental schedule. Male DBA/1J mice (8 weeks old) were immunized by intradermal injection with 100 μg of bovine type II collagen emulsified with CFA. For assessment of the intraperitoneal inoculation of *L. helveticus* SBT2171, the mice were injected with 100 μL of PBS containing 1 mg of *L. helveticus* SBT2171 cells intraperitoneally three times per week after the first immunization, and daily after the second immunization. For oral administration of *L. helveticus* SBT2171, 300 μL of an NaHCO_3_/PBS solution containing 30 mg of freeze-dried bacterial cells was administered daily after the first immunization.

**FIGURE 2:**
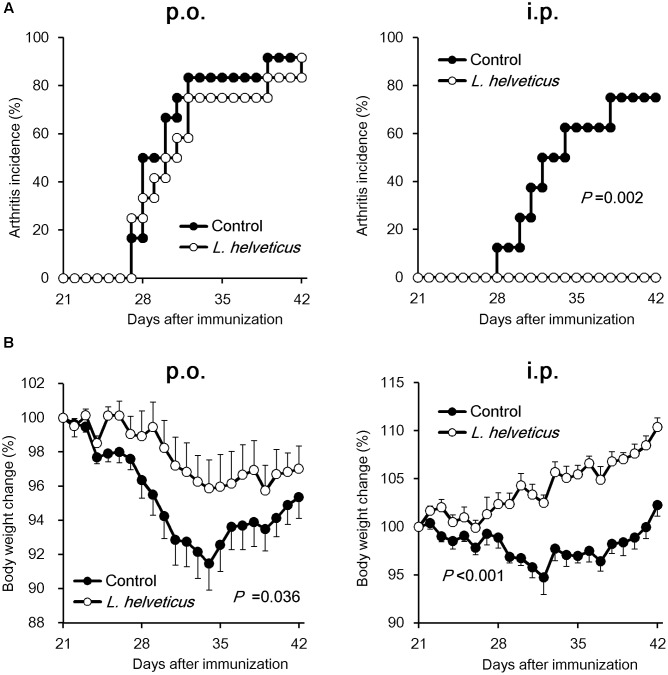
Effects of oral administration and intraperitoneal inoculation of *L. helveticus* SBT2171 on arthritis incidence and body weight loss. CIA was induced in DBA/1J mice by immunization with bovine type II collagen twice, on days 0 and 21. Mice were given *L. helveticus* SBT2171 or vehicle control orally (*per os*; p.o.) (left) or intraperitoneally (i.p.) (right). Arthritis incidence **(A)** and changes in body weight **(B)** were monitored after the second immunization. Data are presented as the mean ± SEM (*n* = 8–12). The data of the group given *L. helveticus* SBT2171 were compared to those of the control group by a log-rank test **(A)** or two-way repeated-measures analysis of variance (group × time interaction) **(B)**.

**FIGURE 3:**
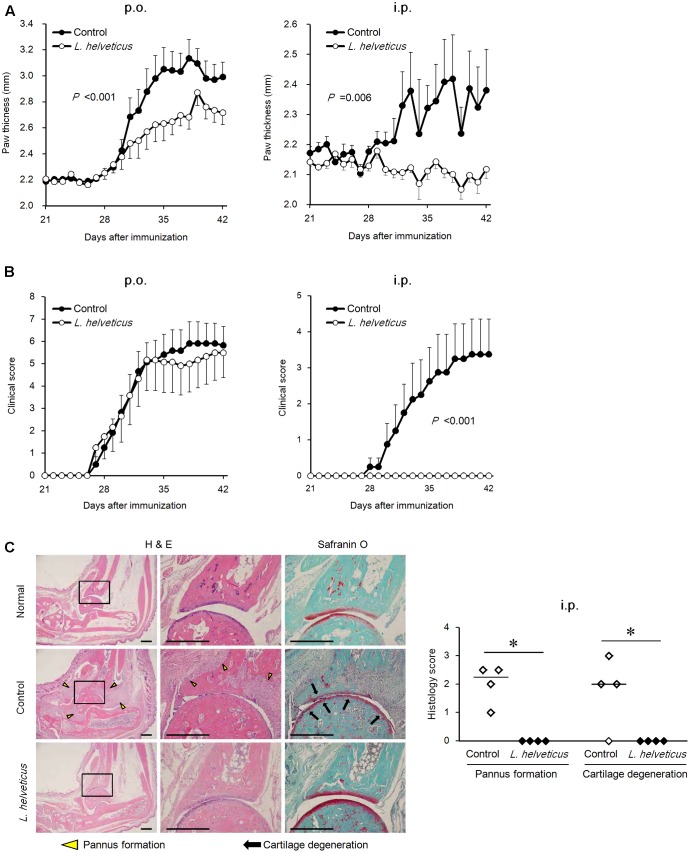
Effects of oral administration and intraperitoneal inoculation of *L. helveticus* SBT2171 on arthritic symptoms. Hind paw thickness **(A)** and clinical score **(B)** were determined in CIA mice given *L. helveticus* SBT2171 orally (p.o.) (left) or intraperitoneally (i.p) (right). Data are presented as the mean ± SEM (*n* = 8–12). **(C)** Histology of the joints of normal and CIA mice receiving vehicle only (control) or intraperitoneal inoculation of *L. helveticus* SBT2171 at day 42. Representative H&E and Safranin O images (left) and histology scores (right) are shown. Bars = 500 μm. Data are presented as dot plots (*n* = 4) and the line represents the median. Data between groups were compared by two-way repeated-measures analysis of variance (group × time interaction) **(A,B)** or the Mann–Whitney *U*-test (^∗^*P* < 0.05) **(C)**.

We examined the efficacy of the intraperitoneal inoculation and oral administration of *L. helveticus* SBT2171 to attenuate CIA in mice. DBA/1J mice were immunized with an emulsion of bovine CII and CFA, and after 21 days, the mice were re-immunized. The experimental schedules are shown in **Figure 1**. The intraperitoneal inoculation of *L. helveticus* SBT2171 suppressed the incidence of arthritis and the increase of clinical score, and decreased body weight loss and hind paw thickness when compared to the control (vehicle-only inoculation) CIA mice (**Figures 2**, **3A,B**). These results were in agreement with our previous report (Hosoya et al., 2014). In addition, histological examination of the joints showed inflammation and cartilage damage in the paws and ankles of the control CIA mice, which were not present in the mice inoculated with *L. helveticus* SBT2171 (**Figure 3C**).

In the oral administration group, the mice were given *L. helveticus* SBT2171 in a sodium bicarbonate (NaHCO_3_) solution (to neutralize and reduce the effect of stomach acid) or NaHCO_3_ solution only as a vehicle control. Oral administration of *L. helveticus* SBT2171 significantly suppressed the CIA symptoms observed in the control mice, such as the body weight loss and hind paw thickening, whereas the incidence of arthritis and the clinical score were not significantly reduced, in contrast to the results for the intraperitoneal inoculation group (**Figures 2**, **3**). We conducted the same experiments in duplicate for both the oral administration and intraperitoneal infection of *L. helveticus* SBT2171. In an independent experiment for oral administration, we also demonstrated an effect of *L. helveticus* SBT2171 on decreasing the body weight loss and paw patch thickness. These results for intraperitoneal infection are similar to those reported in our previous paper (Hosoya et al., 2014).

### *Lactobacillus helveticus* SBT2171 Reduced the Serum Level of CII-Specific Antibodies

**FIGURE 4:**
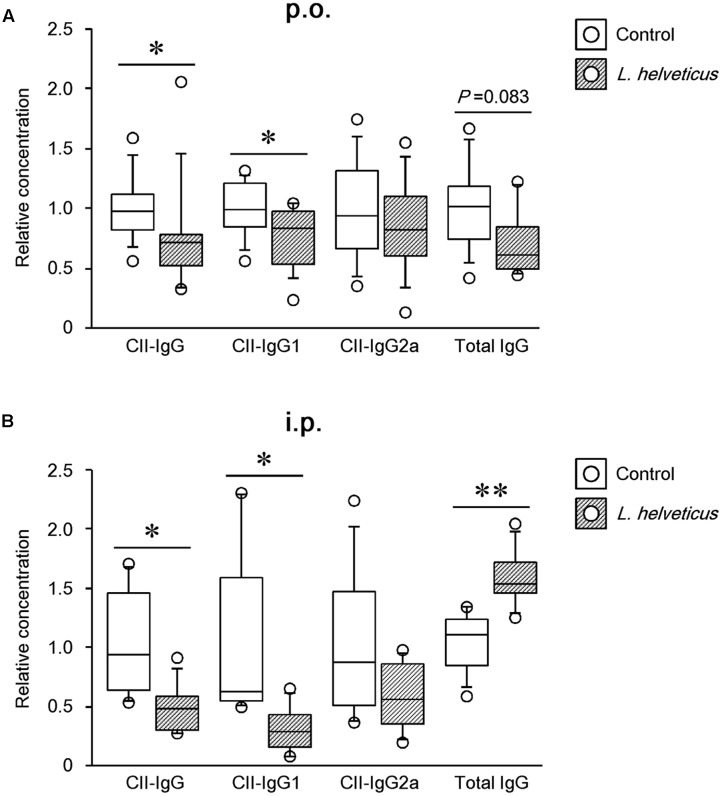
Effects of *L. helveticus* SBT2171 on the serum level of CII-specific IgG and total IgG. The level of antibodies in the serum of CIA mice given *L. helveticus* SBT2171 orally (p.o.) **(A)** or intraperitoneally (i.p.) **(B)** was measured at day 42. Data are presented as dot plots (*n* = 8–12); center line, median; box, 25th–75th percentile of the data; error bars, 10th–90th percentile of the data. ^∗^*P* < 0.05, ^∗∗^*P* < 0.01, compared to the control by the Mann–Whitney *U*-test.

Because anti-collagen antibodies have been shown to play a pathogenic role in CIA (Nandakumar et al., 2003), we first examined the ability of *L. helveticus* SBT2171 to reduce the amount of CII antibodies in the mice. Analysis of serum obtained at day 42 after the first immunization showed a significant reduction in the amount of CII-specific IgG and IgG1 in the mice given *L. helveticus* SBT2171 both orally and intraperitoneally when compared to those in the control mice. However, no significant change in the CII-specific IgG2a level was observed (**Figures 4A,B**). In addition, gene expression analysis (see Supplementary Table S1) showed that the ratio of IL-4 (a Th2 cytokine) to interferon-gamma (IFN-γ, a Th1 cytokine) appeared to be slightly reduced in the LNs of the mice given *L. helveticus* SBT2171 (*P* = 0.093; Supplementary Figure S1). Moreover, the serum level of total IgG (a non-collagen specific antibody) was decreased following oral administration of *L. helveticus* SBT2171.

### Intraperitoneal Inoculation of *L. helveticus* SBT2171 Reduced the Serum IL-6 Level

**FIGURE 5:**
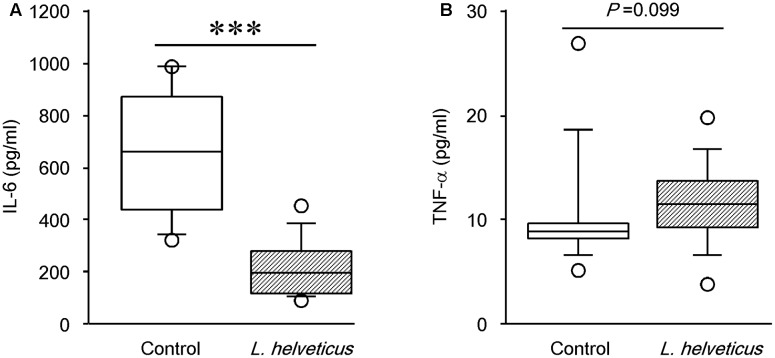
Effects of intraperitoneal inoculation of *L. helveticus* SBT2171 on the serum level of IL-6. The levels of the pro-inflammatory cytokines IL-6 **(A)** and TNF-α **(B)** in the serum of CIA mice given *L. helveticus* SBT2171 or PBS (control) intraperitoneally were measured at day 42 after the first immunization. Data are presented as box plots (*n* = 9–12), as described in the legend to **Figure 4**. ^∗∗∗^*P* < 0.001, compared to the control group by the Mann–Whitney *U*-test.

Several studies have demonstrated that pro-inflammatory cytokines such as IL-6 and TNF-α are involved in joint inflammation and arthritic progression (Choy and Panayi, 2001; Koch, 2005). Therefore, to further investigate the mechanism contributing to the reduction of disease incidence and severity by *L. helveticus* SBT2171, we examined the levels of pro-inflammatory cytokines. The levels of IL-6 and TNF-α in the serum were assessed at day 42 using ELISAs. Compared to the control mice, the IL-6 level was significantly lower in mice inoculated with *L. helveticus* SBT2171 intraperitoneally (**Figure 5**). However, there was no significant difference in the TNF-α level between the *L. helveticus* SBT2171-inoculated and control mice. In contrast, oral administration of *L. helveticus* SBT2171 did not significantly reduce the levels of IL-6 or TNF-α (Supplementary Figure S2A).

### Intraperitoneal Inoculation of *L. helveticus* SBT2171 Decreased the Number of Immune Cells Involved in the Development and Progression of CIA

**FIGURE 6:**
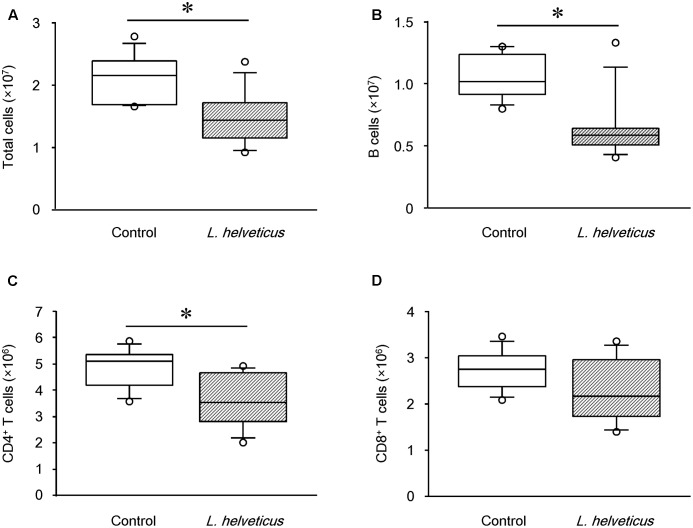
Effects of intraperitoneal inoculation of *L. helveticus* SBT2171 on the number of immune cells in the lymph nodes. Inguinal lymph nodes were collected at day 42 from CIA mice given *L. helveticus* SBT2171 or PBS (control) intraperitoneally. Immune cells were isolated from the lymph nodes and the numbers of total immune cells **(A)**, B cells **(B)**, CD4^+^ T cells **(C)**, and CD8^+^ T cells **(D)** were analyzed by flow cytometry. Data are presented as box plots (*n* = 8). ^∗^*P* < 0.05, compared to the control group by the Mann–Whitney *U*-test.

A variety of immune cells have been implicated in the production of autoantibodies and cytokines, leading to the development of RA and CIA (Choy and Panayi, 2001). To investigate the effect of *L. helveticus* SBT2171 in altering the abundance or function of immune cells, we analyzed cells isolated from the inguinal LNs (the draining LNs in the model) of CIA mice inoculated with *L. helveticus* SBT2171 intraperitoneally using flow cytometry. The numbers of total immune cells, B cells, and CD4^+^ T cells were significantly lower in the inguinal LNs of the mice inoculated with *L. helveticus* SBT2171 when compared to those of the control mice (**Figure 6**). However, there was no significant difference in the number of CD8^+^ T cells. Oral administration of *L. helveticus* SBT2171 did not influence the numbers of inguinal immune cells (Supplementary Figure S2B).

**FIGURE 7:**
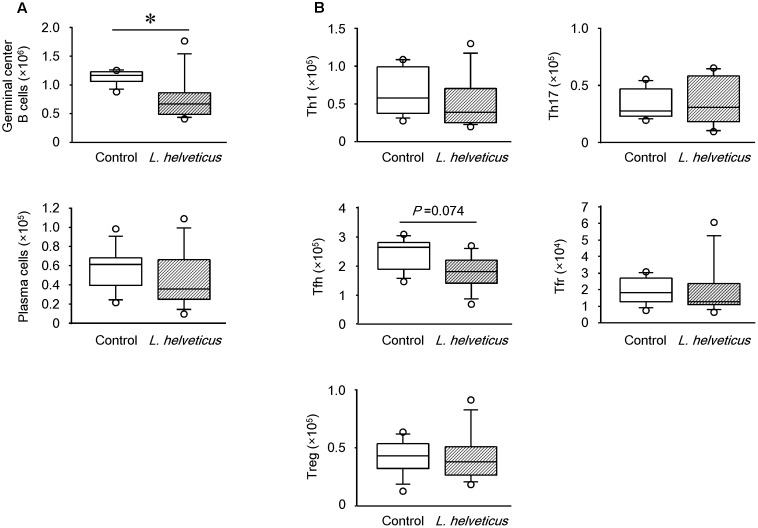
Effects of intraperitoneal inoculation of *L. helveticus* SBT2171 on germinal center B cells and follicular helper T cells in the lymph nodes. Inguinal lymph nodes were collected at day 42 from CIA mice given *L. helveticus* SBT2171 or PBS (control) intraperitoneally. Immune cells were isolated from the lymph nodes and the number of germinal center B cells, plasma cells **(A)**, Th1 cells, Th17 cells, follicular T helper (Tfh) cells, follicular regulatory T cells (Tfrs), and regulatory T cells (Tregs) **(B)** were analyzed by flow cytometry. Data are presented as box plots (*n* = 8). ^∗^*P* < 0.05, compared to the control group by the Mann–Whitney *U*-test.

Upon further analysis of the B cell subsets in the mice inoculated with *L. helveticus* SBT2171, the number of GC B cells, but not plasma cells, was found to be significantly lower than that in control mice (**Figure 7A**). Detailed analysis of the T helper (Th) cell subsets demonstrated a slight reduction of the Tfh cell number in the inguinal LNs of the mice inoculated with *L. helveticus* SBT2171 when compared to that of the control mice, although this reduction was not statistically significant (*P* = 0.074; **Figure 7B**). The numbers of Th1 and Th17 cells, pro-inflammatory Th cell subsets that have been implicated in the development of arthritis (Afzali et al., 2007), were not reduced by the inoculation of *L. helveticus* SBT2171. The numbers of Tregs and Tfrs, which are anti-inflammatory Th cell subsets (Vanderleyden et al., 2014), were also comparable between the mice inoculated with *L. helveticus* SBT2171 and the control mice.

## Discussion

We recently reported that the intraperitoneal inoculation of *L. helveticus* SBT2171 suppressed inflammatory arthritis in CIA mice (Hosoya et al., 2014). In the present study, we conducted a more detailed analysis of the action of *L. helveticus* SBT2171 to reduce or prevent the incidence and progression of CIA, and further investigated the effects of the oral administration of *L. helveticus* SBT2171. The results showed that oral administration could also significantly relieve the joint swelling and suppress the body weight loss caused by CIA, and that intraperitoneal inoculation and oral administration of *L. helveticus* SBT2171 might suppress CIA symptoms through several common mechanisms.

Previous reports have demonstrated the effects of LAB treatment in murine models of RA (Kato et al., 1998; Kano et al., 2003; Amdekar et al., 2011; Kim et al., 2015). The *Lactobacillus casei* strain Shirota reduced the development of CIA and the level of CII-specific antibodies in the serum (Kato et al., 1998). Another *L. casei* strain was also shown to alleviate CIA (Kato et al., 1998). *L. helveticus* HY7801 was shown to prevent the development of CIA, and limit disease progression and severity by reducing antigen-specific IgG levels and the inflammatory immune response (Kim et al., 2015). Similar effects were also observed upon treatment with LAB-containing products. For example, skim milk fermented with *Lactobacillus delbrueckii* ssp. *bulgaricus* OLL1073R-1 also suppressed CIA symptoms and the secretion of pro-inflammatory cytokines (Kano et al., 2003). Despite numerous reports showing the efficacy of LAB, the underlying mechanism of action is yet to be fully determined.

RA is a complex systemic autoimmune disorder characterized by chronic synovitis, leading to the progressive destruction of the joints. The beneficial effects of therapeutic B cell depletion have been demonstrated in RA patients and CIA mice (Svensson et al., 1998; Edwards et al., 2004; Cohen et al., 2006; Yanaba et al., 2007), suggesting the critical role of B cells in the pathogenesis of RA and CIA. Other studies have also demonstrated the presence of lymphoid follicle-like structures in the synovial tissues of RA patients (Weyand and Goronzy, 2003) and CIA mice (Endo et al., 2015). Furthermore, the elevation of autoantibodies such as the anti-cyclic citrullinated peptide antibody in the serum of RA patients and anti-CII antibody in the serum of RA patients and CIA mice has been observed (Svensson et al., 1998; Cohen et al., 2006; Yanaba et al., 2007; Mullazehi et al., 2012). Intravenous administration of anti-CII antibody triggered arthritis-like symptoms in mice (Nandakumar et al., 2003). Together, these findings suggest that the generation of lymphoid follicles and GCs, and the subsequent production of autoantibodies are important drivers of RA and CIA pathogenesis. The GCs are small structures within the follicles of secondary lymphoid organs where B cells proliferate, differentiate, generate the diverse antibody repertoire, and undergo antibody class switching during the immune response to various antigens (Vinuesa et al., 2009; Endo et al., 2015). To form and maintain GCs, Tfh cells are required in the lymphoid organs (Vinuesa and Cyster, 2011), which regulate the differentiation of GC B cells into plasma and memory B cells. During the development of autoimmune diseases, including RA, CD4^+^ T cells (especially Tfh cells) and B cells are essential for the production of autoantibodies. Several reports have demonstrated that these immune cells promoted the proliferation, maturation, and antibody production of B cells in the lymphoid follicles and GCs (Leavenworth et al., 2013). In addition to RA, these cells have also been implicated in other autoimmune disorders such as systemic lupus erythematosus (King et al., 2008; Linterman et al., 2009; Simpson et al., 2010).

In this study, *L. helveticus* SBT2171 given orally or intraperitoneally was shown to suppress the incidence, worsening of disease symptoms (including joint damage and body weight loss), and serum level of anti-CII antibodies in CIA mice. In addition, intraperitoneal inoculation of *L. helveticus* SBT2171 also decreased the number of total B cells, GC B cells, CD4^+^ T cells, and tended to decrease Tfh cells in the draining LNs. Moreover, CII-IgG1 was downregulated following intraperitoneal inoculation of *L. helveticus* SBT2171. IgG1 has been reported to be more important than other IgG subclasses in the development of CIA (Nandakumar et al., 2003; Thorvaldson et al., 2006). Thus, this downregulation might contribute to the preventive effect of *L. helveticus* SBT2171 on CIA symptoms. IgG1 is typically produced during the Th2 immune response (Kato et al., 1998); thus, the present results suggest the ability of *L. helveticus* SBT2171 to suppress Th2 cell activation. However, the total IgG increased following *L. helveticus* SBT2171 injection, which might reflect a general immunoreaction.

These findings could suggest that *L. helveticus* SBT2171 might inhibit the proliferation of B cells (including antibody-producing GC B cells) and CD4^+^ T cells (including Tfh cells, which assist with antibody production), leading to the suppression of follicular formation and decreased autoantibody production, ultimately attenuating the inflammatory symptoms associated with arthritis in CIA mice.

Substantial progress has been made in the development of several anti-RA drugs that target B and T cells, as well as pro-inflammatory cytokines such as TNF-α and IL-6 (Choy and Panayi, 2001; Koch, 2005). IL-6 is involved in multiple immunological processes, including T cell activation, B cell proliferation, initiation of the acute-phase response, and osteoclast differentiation, which all contribute to joint destruction. Tocilizumab, a humanized monoclonal antibody targeting the IL-6 receptor, has been approved for the treatment of RA. Clinical studies on tocilizumab suggested the role of IL-6 in several processes that lead to the development of RA (Fujimoto et al., 2008; Nakashima et al., 2014). In the current study, we showed that intraperitoneal inoculation of *L. helveticus* SBT2171 significantly decreased the level of IL-6 in the serum of CIA mice. In a previous *in vitro* study, we showed that *L. helveticus* SBT2171 was able inhibit the production of inflammatory cytokines, including IL-6, in primary murine immune cells (Yamashita et al., 2014). Other studies have also reported the suppressive effects of LAB on inflammatory cytokine production *in vitro* and *in vivo* (Griet et al., 2014; Novotny et al., 2015). Based on these findings, the reduction in the IL-6 level by *L. helveticus* SBT2171 might closely correlate with CIA alleviation. It is suggested that *L. helveticus* SBT2171 might prevent or suppress CIA symptoms by downregulating the production of IL-6 in immune cells (such as macrophages and dendritic cells) or synoviocytes, the major IL-6-producing cells.

The results of our study suggest that the preventive effect induced by oral administration of *L. helveticus* SBT2171 is weaker than that induced by intraperitoneal inoculation, since the oral administration could suppress the symptoms of CIA such as hind paw thickening and body weight loss, but did not decrease the CIA incidence compared to the control group. One possibility for this difference in the effectiveness of the two administration routes is that the active components of *L. helveticus* contributing to the suppression of CIA might be digested in the stomach or intestine to become inactive, or might be poorly absorbed from the gastrointestinal tract. In fact, the bacterial components oligodeoxynucleotides were found to be altered by the actions of gastric juice and digestive enzymes, resulting in the loss of their immunostimulatory activity (Wang et al., 2015).

In our previous *in vitro* study, *L. helveticus* SBT2171 suppressed the proliferation of primary murine B and T cells stimulated with LPS (Hosoya et al., 2014), suggesting that *L. helveticus* SBT2171 should have activity to suppress the expansion of immune cells, if it could directly interact with them. In the present study, the intraperitoneal inoculation of *L. helveticus* SBT2171 was significantly effective to inhibit the onset and symptoms of CIA. By contrast, oral administration of *L. helveticus* SBT2171 did not significantly downregulate the serum level of IL-6 and the number of T and B cells in the inguinal LNs; although it reduced the amount of the CII-specific antibody and suppressed CIA symptoms, its preventive effect was milder than that of the intraperitoneal inoculation. These results suggest that *L. helveticus* SBT2171 or its bioactive components could reach the inguinal LNs, in which immune cells are activated, through intraperitoneal inoculation, and thereby regulate the number of immune cells. By contrast, fewer of these components would reach the inguinal LNs through an oral administration route, owing to their partial inactivation via digestion when passing though the stomach and intestines.

Given the above findings and the overall differences and similarities in the effects of intraperitoneal inoculation and oral administration, our results suggest a novel potential action of *L. helveticus* SBT2171, in which reduction of both the IL-6 level and pathogenic immune cell numbers might be necessary to suppress or prevent the incidence and reduce the clinical score of CIA.

## Conclusion

Our study demonstrated that both oral administration and intraperitoneal inoculation of *L. helveticus* SBT2171 significantly suppressed the inflammatory symptoms associated with CIA, and the intraperitoneal inoculation further decreased the levels of anti-CII antibodies and the inflammatory cytokine IL-6 in the serum, as well as the abundance of CIA-exacerbating immune cells such as B and T cells. Thus, the administration of *L. helveticus* SBT2171 might be useful to exert a preventive effect for patients with autoimmune diseases, including RA.

## Author Contributions

MY, KM, TE, KU, TH, HN, FS, and TM designed the research. MY, KM, YM, TH, and FS performed the experimental work and analyzed the data. MY, KM, and TM wrote the manuscript.

## Conflict of Interest Statement

MY, KM, KU, TH, and FS are employees of Megmilk Snow Brand Co., Ltd. The other authors declare that the research was conducted in the absence of any commercial or financial relationships that could be construed as a potential conflict of interest.

## Abbreviations

CFA: Complete Freund’s Adjuvant
CIA: collagen-induced arthritis
CII: type II collagen
CII-IgG: type II collagen-specific IgG
GC: germinal center
IL: interleukin
LAB: lactic acid bacteria
LNs: lymph nodes
LPS: lipopolysaccharide
RA: rheumatoid arthritis
Tfh: follicular T helper
Tfrs: follicular regulatory T cells
Th: T helper
Tregs: regulatory T cells
