# Supplementary material for: Preventive Effect of Lactobacillus helveticus SBT2171 on Collagen-Induced Arthritis in Mice
Source: Front Microbiol. 2017 Jun 21;8:1159. doi: 10.3389/fmicb.2017.01159 (PMC5478730; doi:10.3389/fmicb.2017.01159)
Supplement: Supplementary file 1 [file Data_Sheet_1.DOC]

Supplementary Material

# Preventive effect of Lactobacillus helveticus SBT2171 on collagen-induced arthritis in mice

Maya Yamashita, Kurumi Matsumoto, Tsutomu Endo, Ken Ukibe, Tomohiro Hosoya, Yumi Matsubara, Hisako Nakagawa, Fumihiko Sakai, Tadaaki Miyazaki*

*** Correspondence:** Tadaaki Miyazaki: [miyazaki@pop.med.hokudai.ac.jp](mailto:miyazaki@pop.med.hokudai.ac.jp)

**Supplementary Methods**

***Gene expression analysis***

Total RNA was isolated from the inguinal LNs of CIA mice using the TRIzol reagent (Life Technologies, Thermo Scientific, Waltham, MA, USA). RNA was reverse-transcribed into complementary DNA using the ReverTra Ace qPCR kit (TOYOBO, Osaka, Japan). Real-time PCRs were performed using the KAPA SYBR FAST Universal qPCR Kit (KAPA BIOSYSTEMS, Boston, MA, USA) according to the manufacturer’s instructions. Sequences of the PCR primers are shown in Supplementary Table 1. Data were normalized to *Gapdh* gene expression.

**Supplementary Figures**


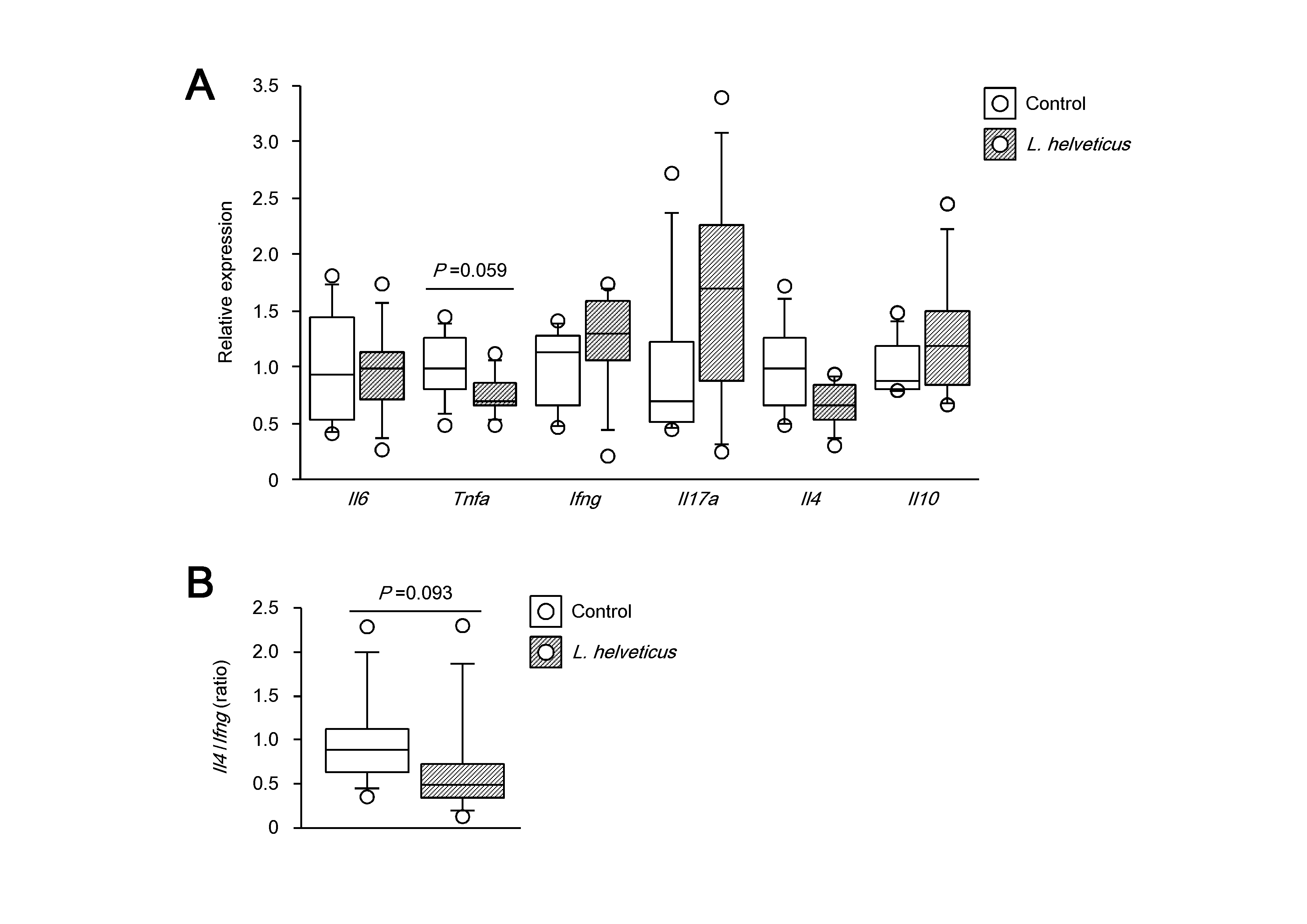


**Supplementary Figure S1**

**Effects of intraperitoneal inoculation of *L. helveticus* SBT2171 on cytokine gene expression levels and the ratio of Th2 and Th1 cytokines in the lymph nodes of CIA mice.**

The inguinal lymph nodes of CIA mice given *L. helveticus* SBT2171 intraperitoneally or PBS only (control) were collected at day 42. Relative cytokine gene expression levels (**A**) and the ratio of *Il4* to *Ifng* (**B**) were determined. Data are shown as box plots (n = 8). The data of the group inoculated with *L. helveticus* SBT2171 were compared to those of the control group using the Mann–Whitney U test.

**
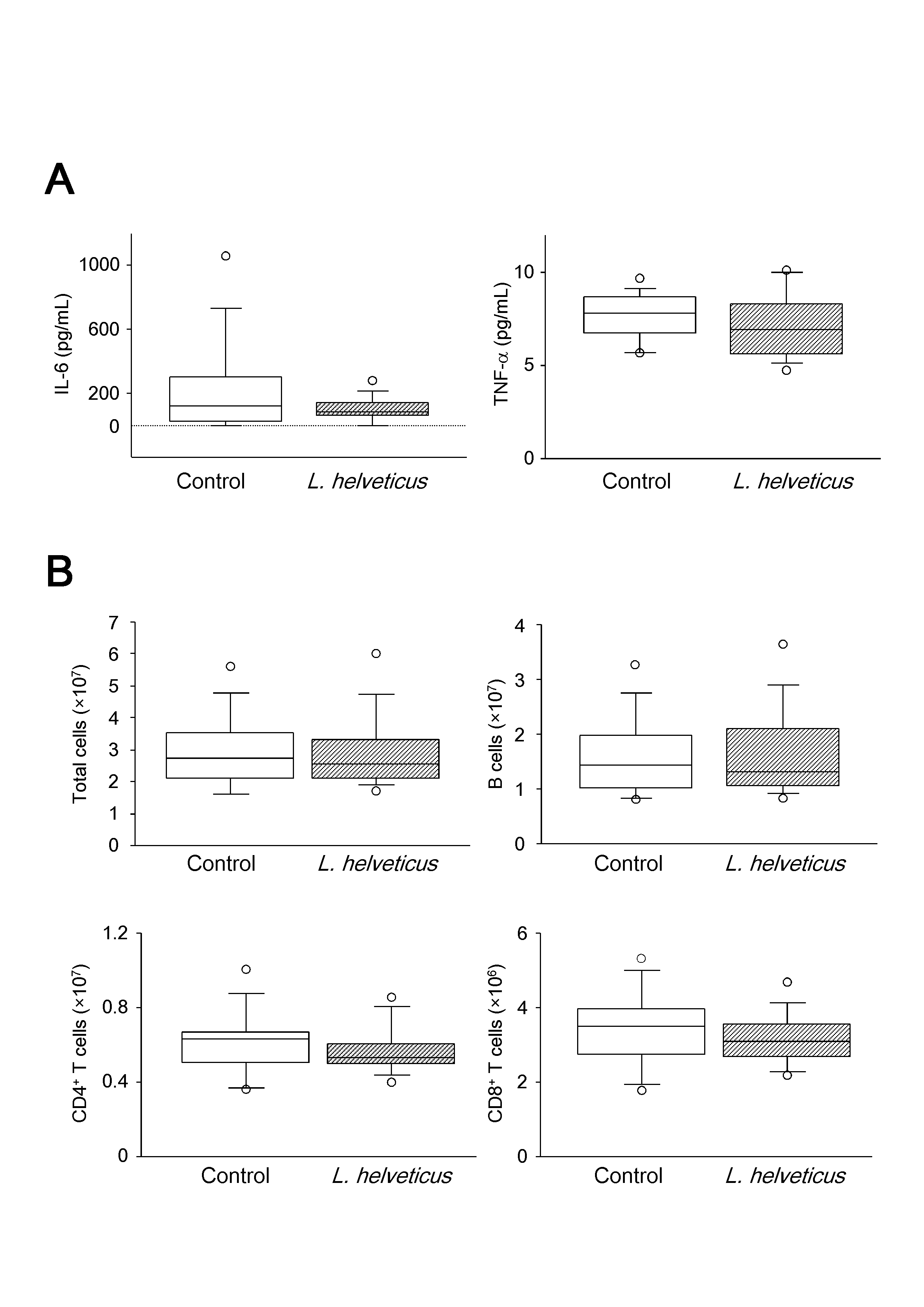
**

**Supplementary Figure S2**

**The serum levels of IL-6 and TNF-α and the numbers of immune cells in the lymph nodes of the mice orally administered *L. helveticus* SBT2171.**

The levels of the pro-inflammatory cytokines IL-6 and TNF-α in the serum (**A**) and the numbers of total immune cells, B cells, CD4^+^ T cells, and CD8^+^ T cells in the inguinal lymph nodes (**B**) of CIA mice given *L. helveticus* SBT2171 or PBS (control) orally were measured at day 42 after the first immunization. Data are presented as box plots (n = 12), as described in the legend to Fig. 4.

**Supplementary Table**

**Supplementary Table 1.** Primers used in the gene expression analysis.

| Gene Symbol | Forward Primer 5′→3′ | Reverse Primer 5′→3′ |
| --- | --- | --- |
| *Gapdh* | AAGGGCTCATGACCACAGTC | GGATGCAGGGATGATGTTCT |
| *Il6* | CGTGGAAATGAGAAAAGAGTTGTGC | TGGTACTCCAGAAGACCAGAGGA |
| *Tnfa* | AGCCCACGTCGTAGCAAACCAC | CGGGGCAGCCTTGTCCCTTG |
| *Ifng* | CTGCAGAGCCAGATTATCTC | CCTGTGGGTTGTTGACCTCA |
| *Il17a* | GCTCCAGAAGGCCCTCAGA | CTTTCCCTCCGCATTGACA |
| *Il4* | CGAGCTCACTCTCTGTGGTG | TGAACGAGGTCACAGGAGAA |
| *Il10* | GCCCCAGGCAGAGAAGCATGG | GGGGAGAAATCGATGACAGCGCC |
